# A Useful Antiseptic

**Published:** 1904-05-15

**Authors:** Bernard Bennette


### A USEFUL ANTISEPTIC.

BY BERNARD BENNETTE, L.D.S., EDIN.

Some of the qualities required in an antiseptic for dental use are as follows: 1st, it must be non-escharotic; 2d, non-irritant; 3d, should have localized action; 4th, should be reasonably non-toxic; 5th, should not discolor the dentine of teeth in which it is used; and 6th, should not coagulate albumin. Now take one or two of the more common agents used as antiseptics. Perchloride of mercury, for instance, is a deadly poison, it is an irritant, it coagulates albumin forming insoluble mercuric albuminate, discolors tooth substance by the formation of black sulphide of mercury, finally it has a most disagreeable metallic taste, and should not be used as an ingredient of a mouth wash, except when specially indicated, as in oral syphilis. Formaldehyde—as formalin forty percent solution—*may* be used *with extreme care* in the treatment of pulpless teeth. It is excellent as a mouth wash in one percent or two percent solution, but if retained too long in the mouth, causes a peculiar biting sensation. It is highly irritant and necrotic. The essential oils are contra-indicated in anterior teeth, with the exception perhaps of oil of cloves, owing to the fact that they discolor the dentine. Iodoform and creosote are abominations. The antiseptic which I wish to bring before your notice is a simple one; it is a modification of camphenol or campho-phenique. To prepare it, take two fluid ounces of pure liquid carbolic acid and equal parts camphor, menthol and thymol. Place the latter substances in a colored three-ounce bottle, pour the acid over and shake. It immediately becomes milky, complete solution takes two or three days, more menthol, camphor and thymol should be added till acid is saturated. Decant clear solution and it is ready for use. It should be kept in a brown or dark blue bottle with a well-fitting glass stopper. Indications:

A pulpless tooth with chronic abscess; gain access to canal, syringe out with $H_2O_2$, three percent, treat with sodium dioxide, dry canal and flood with antiseptic, forcing it into the abscess sac and out through sinus, if one be present. The apex is now sealed, and subsequent treatment is done through the sinus, one drop of the agent being placed in the sac by means of a hypodermic syringe. Sometimes one, but generally two or three sittings are necessary to bring about a cure; it is not good treatment to hurry with a chronic abscess. It is useful used in full strength, for wiping out cavities prior to filling. For pulpitis it is valuable. A small pledget of cotton-wool moistened with it, placed over the pulp and covered with a light dressing, inserted without pressure, will give immediate relief. Four minims in half a tumbler of warm water makes a good mouth wash, leaving behind an agreeable sensation of cleanliness. It is, however, not recommended as a mouth wash for constant use, but mainly after extractions or operations in the mouth. It is an efficient agent for the relief of pain after the extraction of teeth with periodontitis. I regard it as next in value as an all-round antiseptic and germicide to hydrogen peroxide. It is very simple to make; the addition of saccharine gr. v. to every ounce makes it more acceptable to patients as a mouth wash.—*Dental Record.*

